# Prevalence and presentation of hyperdontia in a non-syndromic, mixed Nigerian population

**DOI:** 10.4317/jced.55767

**Published:** 2019-10-01

**Authors:** Seidu Bello, Wisdom Olatunbosun, John Adeoye, Abiodun Adebayo, Nathan Ikimi

**Affiliations:** 1 Department of Dental and Maxillofacial Surgery, State House Medical Centre, Aso Rock, Asokoro, Abuja, Federal Capital Territory, Nigeria; 2 QH Specialist Dental Clinics and Research Centre, Gwarinpa, Abuja, Federal Capital Territory, Nigeria; 3 Research division, Cleft and Facial Deformity Foundation, International Craniofacial Academy, Gwarinpa, Abuja, Federal Capital Territory, Nigeria

## Abstract

**Background:**

Although there have been studies on the prevalence and pattern of hyperdontia in sub-Saharan African subjects with similar cultural backgrounds, based on our search, none have been able to consider these epidemiological parameters in a multiethnic black population, which is expected to add substantial knowledge to what is available.

**Material and Methods:**

This is a retrospective study on the panoramic radiographs of subjects who presented at two dental centres in Abuja, Nigeria between June 2013 and June 2018. Radiographic interpretations were carried out by three independent observers, trained on computer assisted radiographic image interpretation. Data were collected and analyzed using Statistical package for the Social sciences (SPSS) version 22 (IBM Corp, Armonk, USA).

**Results:**

One thousand eight hundred and thirty seven (1837) panoramic radiographs were studied. Subject comprised males and females between ages 12 – 95 years with an average of 35.0 years. The prevalence of unilateral hyperdontia was 1.47% while an occurrence rate of 0.27 was observed for bilateral and multiple hyperdontia. For maxillary hyperdontia, a prevalence of 1.09% was recorded which was significantly more common than the mandibular type (0.65). Of note is that all the supernumerary teeth types were commonly observed in the maxilla except the parapremolar type, with a mandibular occurrence rate of 76.9%.

**Conclusions:**

From this study, we can conclude that the prevalence of hyperdontia (across different black ethnicities) is low. Although, follicular epithelium around the tooth and root resorption of the enlargement around the adjacent teeth was observed, most were asymptomatic (87.0%) and required no intervention.

** Key words:**Hyperdontia, panoramic radiographs, Sub-Saharan Africa, supernumerary teeth.

## Introduction

Supernumerary teeth are extra teeth found in the dental arches in addition to the normal number of the adult dentition. The condition in which these additional teeth are present is termed hyperdontia which represents one of the types of dental anomalies involving tooth number (the other being hypodontia/congenitally missing teeth) (1). Conventionally, supernumerary teeth are defined as odontogenic structures formed from a tooth germ that in excess of its usual number in any of the dental arch quadrants (1). They may be seen in either the primary or permanent dentition; although, their occurrence is more common in the later (2), which may be unilateral or bilateral in one or both jaws. In more severe presentations, their occurrence in both jaws may be multiple, with this frequently occurring in syndromes associated with supernumerary teeth including – Cleidocranial dysplasia (3), Gardner’s Syndrome (4), Incontinentia Pigmenti (5) and other notable syndrome associated with orofacial cleft (6).

The etiology of supernumerary teeth is still not fully understood, although both genetic and environmental factors have been implicated. There are various theories that have been proposed to explain the development of different supernumerary teeth (7-10). One of such theories is the ‘dichotomy theory’ which suggests that a single tooth bud may divide into two resulting in the development and formation of two symmetric or asymmetric teeth (7). Furthermore, the type of teeth formed is dependent on the mode of division of the tooth germ; if the tooth bud separates into two equal halves, this result in the formation of two eumorphic teeth with the additional one termed a “supplemental tooth” (11). However, if the tooth bud divides into two unequal parts, the supernumerary teeth will be different in size and morphology (heteromorphic tooth). Another prominent aetiologic hypothesis is the ‘atavism theory’ (8-9), which allude the development of supernumerary teeth to retrogression of the present number of dentition to ancestral forms comprising larger number of teeth. Currently, the most accepted theory of supernumerary teeth formation is the ‘dental lamina hyperactivity theory’ (10) which suggests that hyperactivity of the dental lamina or its remnant rest cells following induction by initiation factors results in the development of the dental papilla and subsequently, an enamel organ that matures into a supernumerary tooth. In this theory, a eumorph is formed from the lingual extension of an additional tooth bud while a heteromorphic tooth is formed from the hyperactivity of a dental lamina remnant (9).

Supernumerary teeth can be classified based on different parameters. Recent update of literature classifies them according to four – morphology, location, position and orientation (6). Based on form, they are classified as conical, tuberculate, supplemental and odontomes (11) while depending on their location, they are classified as mesiodens, distomolar, paramolar and parapremolars (12). Their position within the jaw varies as they may be placed labially, buccally or palatally with varying orientations (vertical, horizontal or inverted) (6). The clinical implications of supernumerary teeth vary according to their type (based on the aforementioned classifications), dentition affected, presence or degree of their impaction as well as other associated factors. These implications range from being clinically asymptomatic to complications that may affect the by-standing normal teeth such as crowding, failure or delayed tooth eruption, midline diastema, dental caries, fracture and root resorption which may warrant their removal (13).

Hyperdontia, which represents about 1 – 3% of all dental anomalies (1), has a prevalence that ranges between 0.1-3.8% in permanent dentition and 0.35-0.6% in primary dentition (14,15). Variations in the prevalence of supernumerary teeth are associated with demographic factors, notably gender and race with lower prevalence rates reported in Caucasians (8) and comparatively higher prevalence rates in Mongoloids (16) and individuals of African descent (17). The commonest site of occurrence is the anterior midline of the maxilla, with the maxillary molar area being the second most common area of occurrence (6).

Nigeria is a West African nation comprising individuals of diverse ethnicity, culture and religion, with each ethnic group resident in their respective sub-regions. Abuja, which is the capital city of Nigeria, located in her north central sub-region was created to encompass all individuals irrespective of their ethnic or religious backgrounds (18). As such, this epidemiological survey conducted in the Federal capital territory is borne out of the need to determine the prevalence of supernumerary teeth in a multiethnic, Nigerian population which is unique in itself as most studies that have emanated from Sub-Saharan Africa have been conducted among individuals of similar ethnicity which may have introduced selection bias ultimately limiting the ability to externally generalize their findings (19). In addition, this study aims to serve as a source of baseline information for dental professionals and add meaningful evidence to the body of literature at large.

## Material and Methods

This is a retrospective study that was carried out at the dental clinics in two centres in Abuja, Nigeria – State House Medical Centre (Public facility) and QH Specialist Dental Clinics and Research Centre (Private facility). Digital copies of panoramic radiographs in addition to the manual health records of dental patients in both health facilities from June 2013 to June 2018 were obtained. These radiographs and their parent data were assessed and included into the study using the following criteria:

• Male and female individuals of Nigerian origin, resident in Abuja

• Adolescents and Adults above 12 years of age with complete dentition

Exclusion criteria included:

• Individuals with incomplete health records or radiographically apparent retained deciduous tooth/teeth

• Reported evidence of associated systemic pathologies in health records

• Lack of radiographic clarity or presence of artifacts

• Presence of radiographic evidence of pathological conditions such as cysts, neoplasms and fracture

• Presence of osseointegrated, maxillofacial implants or orthodontic appliances

The OPGs in both institutions were obtained with the ORTHOPHOS XGPlusDS/Ceph (Sirona Dental systems GmbH, Bensheim, Germany) digital panoramic machine (Tube voltage: 60-90kV, Tube current: 3 – 16mA, Total filtration of X-ray tube assembly: >2.5mm and magnification coefficient: 1.25) while tools of the SIDEXIS radiographic imaging software (Bensheim, Germany) were used to enhance image clarity during the analysis process. Three independent observers (WO, JA and AA) with prior training in computer assisted radiographic image interpretation evaluated each radiograph for the presence or absence of supernumerary teeth; their position, orientation, shape, eruption status, developmental stage, as well as the presence of associated pathology. Inter-observer reliability was substantial with a Cohen’s Kappa (ᵏ) value of 0.76. Ethical approval for the study was obtained from the institution’s ethics review and informed consent was obtained from all participants before inclusion into the study.

-Statistical Analyses

The data collected were analyzed using the Statistical Package for the Social Sciences (SPSS) version 22 (IBM Corp, Armonk, USA). Descriptive statistics for the socio-demographic characteristics of the included subjects were obtained. The prevalence of this condition was determined by simple division methods, taking the whole sample of included subjects (population at risk) as the denominator and the number of subjects with at least one supernumerary tooth as the numerator. Normality of qualitative and continuous distributions was ascertained using Kolmogrov-Smirnov test while the Mann Whitney U test was employed to determine their statistical difference. Associations between categorical variables were determined using the Pearson’s chi-square test. Level of significance for all statistical tests was set at *p*<0.05.

## Results

A total of 3616 orthopantomograms were obtained from both institutions, and only 1837 of these were finally included in the study based on the criteria listed above in the ‘methods’ section. The age of subjects ranged between 12 – 95 years with a mean of 35.0 + 16.04 years. Most subjects whose radiographs were studied were males, with females accounting for 36.2% of included subjects. Digital analysis of the panoramic radiographs revealed that a total of 32 subjects had at least one supernumerary teeth present, indicating a prevalence of 1.74%. Furthermore, of all 55801 teeth studied, 46 (0.07%) were supernumerary teeth which were distributed in descending order based on their location as follows: distomolars (n=21,45.6%), parapremolars (n=13, 28.3%), paramolars (n=5, 10.9%), lateral canines (n=4, 8.7%), mesiodens (n=2, 4.3%) and a fifth molar (n=1, 2.2%).

The mean age of subjects with hyperdontia was 36.2 + 13.38 years which was comparable and not significantly higher than the mean age of the total subjects in the study (*p*=0.506).Number of male and female participants were evenly distributed, and supernumerary teeth types (based on location)such as mesiodens, distomolar, paramolar and parapremolars were observed more in males while lateral canines were commoner in females (75.0%), although this distribution was not statistically significant (*p*>0.05) ( Table 1).

**Table 1:**
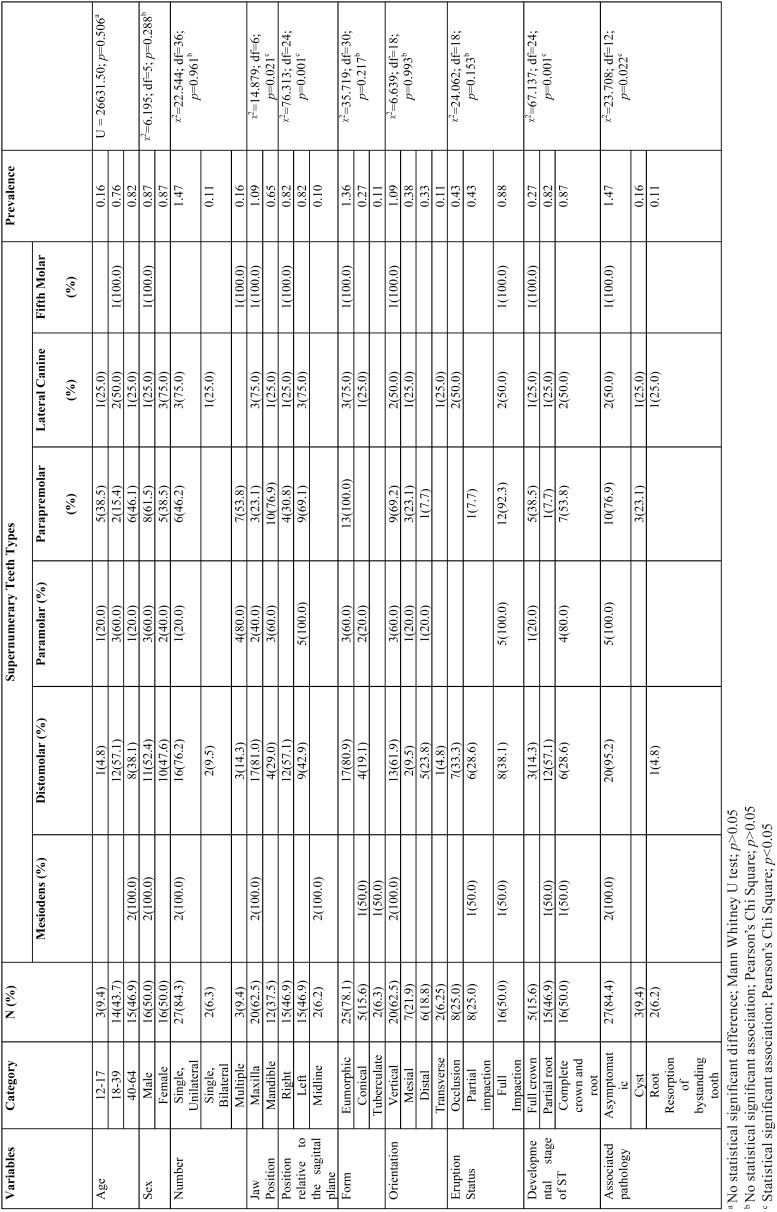
Demography and Characteristics of supernumerary teeth observed in 32 subjects with hyperdontia.

Twenty-seven subjects (84.3%) had unilateral hyperdontia while multiple and bilateral hyperdontia were observed in 9.4% (n=3) and 6.3% (n=2) of total subjects respectively. While most mesiodentes, distomolars and lateral canines were often observed in the panoramic radiographs as lone additions to normal permanent dentition, more paramolars and parapremolars were seen in subjects with multiple supernumerary teeth ( Table 1). Noteworthy is the occurrence of a ‘fifth molar” in one of the subjects with multiple supernumerary teeth which in this case, subject’s health records confirmed it as a non-syndromic occurrence.

Regarding the jaw prevalence of supernumerary teeth, maxillary hyperdontia (1.09) was significantly more common than the mandibular variant of the condition (0.65) (*p*=0.021). Also, all the supernumerary teeth types were commonly observed in the maxilla except the parapremolar type, with a mandibular occurrence rate of 76.9% (n=10).As regards the relationship of the supernumerary tooth to the sagittal plane, only the mesiodens presented in a position along the midline of the maxilla as against others such as distomolars and fifth molars which were common on the right, and paramolars, parapremolars and lateral canines that appear to be more common on the left side ( Table 1). Similarly, the aforementioned positional relationship of the supernumerary teeth relative to the sagittal plane was statistically significant (*p*=0.001).

The morphology of most of the teeth (n=37, 80.4%) were similar to those of their adjacent normal counterparts (eumorphic/supplemental), others were either conical (17.4%) or barrel-shaped (2.2%). Most of the supernumerary teeth assumed a vertical orientation (n=30, 65.2%) while seven (7) teeth were oriented both in the mesial and distal directions respectively. Radiographic observation further revealed that more than half of the supernumerary teeth were fully embedded with the jaw bone (n=29, 63.0%) while 19.4% and 17.6% were at full occlusion and partially impacted respectively (*p*<0.05). Only six (6) supernumerary teeth presented with accompanying radiographic pathology which included cysts (8.7%) and external root resorption of adjacent teeth (4.4%). Parapremolars were the mostly implicated type of supernumerary teeth associated with cyst formation while the presence of external root resorption of the adjacent teeth occurred in a distomolar and lateral canine.

## Discussion

This study was carried out to bring to light the frequency and pattern of presentation of supernumerary teeth in Nigerian subjects of diverse ethnicity, in other to make stronger deductions which would serve as a guide to clinicians locally and globally as regards the congenital dental anomaly. The prevalence of hyperdontia has been described in different populations; employing study designs with data collection via clinical examination of subjects, radiographic analysis or both. The reports of these authors have ranged from lower prevalence rates such as 0.75% in a Middle Eastern population (20), 1.0% in a Turkish population (21), and 1.5% in Swiss (22) and Southern Nigerian population (23) respectively to higher values between 3% - 12.5% in Western Romanian (24), South African (17), North Eastern Chinese (25) as well as South-west and South-south Nigerian populations (26-27). The prevalence of hyperdontia in our radiographic study is 1.74% which falls within the range of occurrence values, based on the observations of the aforementioned authors. Although on the lower end of the set of prevalence values, this finding further buttresses the racial and regional disparity in the occurrence of hyperdontia globally. Even more, this geographical disparity inference of the condition can be drawn to different sub-regions and ethnicities within the same country. For instance, in Nigerian subjects, Adeyemi *et al.* (26) initially reported a prevalence of 9.0% from a study involving the analysis of 100 panoramic radiographs of orthodontic patients in Ibadan, south-west Nigeria while subsequently in Benin City, south-south Nigeria, a significantly lower prevalence of 1.5% was reported (23). In fact, in Abraka within the same south-south region of Nigeria, Anibor *et al.* (27) observed a markedly high prevalence rate of 12.7% from clinical examination of 1004 subjects aged between 18 – 30 years which buttresses the cultural variation of the occurrence of hyperdontia. In this light, our prevalence of 1.74% which has been deduced from the OPG analysis of a mixed population, cuts across the various ethnicities in the region; thus, presenting an occurrence rate that may better characterize the frequency of the dental anomaly and allow for extrapolations to Nigerians and sub-Saharan Africans regardless of their ethno-cultural background. Regarding the sex preponderance of hyperdontia, our study reports an equal prevalence of 0.87 in both males and females which is in contrast with the observations of Mahabob *et al.* (28) and Goksel *et al.* (29) in South India and Turkey respectively despite the similarity in the male to female ratio of the total subjects in all three studies.

Of the 46 supernumerary teeth identified, most of them were distomolars (45.6%) occurring in the maxillary posterior segment of the jaws which is in agreement with more prevalent site of the jaws for distomolars as reported by Kara *et al.* (30) in a Turkish population. However, this finding in our population is unconventional and is in contrast with the reports of Bratu *et al.* (24), Schmuckli *et al.* (22), Fidele *et al.* (25), and Patil *et al.* (2) in Western Romania, Switzerland, Turkey, north east China and north India respectively; all of whose findings were along the more popular notion that supernumerary teeth are more commonly mesiodentes or supplemental lateral incisors in the anterior region of the maxilla. Furthermore, our finding on the prevalent supernumerary tooth type and jaw location is in contrast with other findings by authors - Anibor *et al.* (27) in south-south Nigeria and Al Muheiri *et al.* (20) in a Northern Emirati population where mandibular predominance was reported in the incisor and premolar regions respectively. Although, our study finds supernumerary teeth to be less common in the mandible, most cases of mandibular hyperdontia involved the presence of supernumerary teeth in the premolar region (55.5%). Most of the subjects had unilateral hyperdontia (84.3%) with the distomolars being the most implicated type of supernumerary teeth (57.1%). The single supernumerary tooth in these subjects was more on the left side of both jaws than the right or the midline with a ratio of 12.5:7:1 respectively. This finding based on the number of supernumerary teeth was in agreement with the reports of Al Muheiri *et al.* (20) and Fidele *et al.* (25) and may be adduced to the predominance of bilateral and multiple supernumerary teeth in individuals with other craniofacial or systemic congenital anomalies (6).

An important reason for the use of panoramic radiographs to determine the prevalence of hyperdontia as opposed to clinical examination or analysis of health records solely is due to the non-eruptive nature of some supernumerary teeth which may lead to underestimation of the condition. Our study found that only 19.6% of supernumerary teeth were in occlusion with most being impacted either completely or partially which corroborates the earlier reports of Fidele *et al.* (25) and Al Muheri *et al.* (20). In contrast, this finding in not keeping with the reports of Bratu *et al.* (24) where only 4% of the supernumerary tooth identified following clinical examination and radiographic analysis were impacted. One would expect that with the skewed distribution of supernumerary teeth towards impaction in this study, most of these extra teeth would have been associated with dentoalveolar discrepancies such as altered eruption or development of related tooth, crowding, tooth displacement or even rotation. However, this is not the case as 87.0% of the supernumerary teeth are asymptomatic according to both subjects’ health records and radiographic analyses which may be attributed to the somewhat quiescent nature of the distomolars and paramolars which were the most common types seen in this study as opposed to mesiodens which are more implicated in the aetiology of dentoalveolar discrepancies (31). Only complications related to the enlargement of follicular epithelium around the tooth and root resorption of the adjacent teeth were observed in this study which does not corroborate the findings of several authors (12,25,28) who have cited tooth displacement as the most common complication of supernumerary teeth.

Though unique in the multi-ethnic nature of included subjects, our study is not without limitations. Although all included radiographs had complete permanent dentition, some may have actually been obtained from patients who might have undergone extraction of their supernumerary teeth in the past, which were also not included in their health records. Since subjects could not be recalled, this may have resulted in possible underestimation of the prevalence of hyperdontia and characteristics of supernumerary teeth in this study.

## Conclusions

The prevalence of hyperdontia in this retrospective, radiographic study is 1.74% (approximately 1 in 57 patients), with equal male and female prevalence rates. Distomolars were the most common supernumerary teeth observed in this study. More supernumerary teeth were seen in the maxilla than mandible, although, in the mandible, a preponderance of paramolars and parapremolars were observed. Most of the subjects had unilateral hyperdontia with the supernumerary teeth being common on the left side and four out of every five supernumerary teeth had some form of bony impaction whether full or partial. Although these characterizations of hyperdontia can be applied in other African settings, we recommend that future studies adopt a multinational approach so as to draw even stronger conclusions on the subject matter in the region.
